# The Moderating Effect of Atypical Events on the Relationship Between Heart Rate and Stress in Medical Residents Working in an Intensive Care Unit: Longitudinal, Observational Daily Diary Study

**DOI:** 10.2196/67822

**Published:** 2025-09-05

**Authors:** Ruibei Li, Ujjwal Pasupulety, Wellington Chang, Adam C Frank

**Affiliations:** 1 Los Angeles General Medical Center Los Angeles, CA United States; 2 Department of Psychiatry and the Behavioral Sciences Keck School of Medicine University of Southern California Los Angeles, CA United States

**Keywords:** wearables, medical residents, heart rate, stress, linear mixed models, ecological momentary assessments, intensive care unit

## Abstract

**Background:**

Residency is a critical period in a physician’s training, characterized by significant physical, cognitive, and emotional demands that make residents highly susceptible to stress and associated negative health outcomes. While physiological signals such as heart rate have been explored as potential biomarkers of stress, their predictive utility in high-stress environments such as the intensive care unit (ICU) remains inconclusive, especially when factoring in atypical events that can further exacerbate resident stress levels.

**Objective:**

This study aimed to investigate the relationship between daily average heart rate (AHR) and perceived stress among ICU residents and examine the moderating effect of atypical events on this relationship.

**Methods:**

The TILES (Tracking Individual Performance With Sensors)-2019 dataset collected longitudinal data from 44 ICU residents who provided daily self-reported stress ratings and wore a Fitbit device to track physiological data over a 3-week period. The main predictor variables were AHR and the occurrence of atypical events (both work and life related and daily hassles). The primary outcome was the level of perceived stress measured on a 7-point Likert scale. Linear mixed models were used to analyze the relationship between AHR and stress, accounting for within-subject and between-subject variance. Interaction effects between AHR and atypical events were also examined.

**Results:**

The analysis revealed a significant positive association between AHR and perceived stress (β=0.032; *P*=.04) on standard days. However, this relationship was attenuated by the presence of negative atypical events (β=−0.076; *P*=.02). We further analyzed whether the severity of negative atypical events had an additional moderating effect but found no statistical significance.

**Conclusions:**

AHR is a potential physiological marker for perceived stress in ICU residents, but its effect is moderated by negative atypical events. Future research should replicate these findings in more diverse cohorts, assess their generalizability to broader populations, and control for additional confounding variables. Incorporating negative atypical events into stress assessment could lead to more accurate and context-sensitive interpretations of physiological data.

## Introduction

### Background

For graduating physicians, residency is a period of practical training that is pivotal in determining future success as a health care practitioner [1]. On a daily basis, residents are confronted with challenging medical and professional scenarios that impact personal well-being [2]. The physical, cognitive, and emotional demands placed upon physicians in training make this subpopulation highly susceptible to stress, burnout, imposter syndrome, and various other subclinical and clinical mental health conditions [3-6]. Given the perennially strained health care system and its unceasing requirements for resilient health care professionals, understanding the factors that modulate resident well-being is of paramount importance.

Several studies have ascertained the effectiveness of physiological signals as biomarkers of human emotions, behavioral responses, and stress [7,8]. The commercialization of wearable computing devices (ie, wearables) has allowed for an affordable and unobtrusive mechanism to procure longitudinal physiological data [9]. Smartphone apps offer a convenient apparatus to conduct ecological momentary assessments (EMAs) that instantly capture snapshots of users’ behaviors and experiences via personal devices [10]. This combination of passive and active monitoring yields comprehensive biobehavioral data that can be leveraged to study the relationship between physiological signals and various psychological phenomena within specific populations. One of the first studies to use this methodology on resident physicians identified resilience indicators associated with physiological factors such as sleeping behavior, physical activity, increased mood, and reduced heart rate (HR) and mood variability [11].

Intensive care units (ICUs) present a unique clinical setting where patient cases become particularly stressful due to their urgency and complexity and the potential for rapid deterioration [12]. Apart from fulfilling day-to-day responsibilities (eg, rounds and paperwork), residents working an ICU rotation must be prepared to handle unexpected emergencies that are atypical to their regular workflow. These atypical events, which can range from individual patient deaths [13-15] to mass casualty incidents [16,17], generally overwhelm hospital resources and impair residents’ decision-making due to elevations in stress. Atypical events that are unrelated to work (eg, life events and daily hassles) can also exacerbate resident stress [18].

While multiple studies have used wearables and EMAs to evaluate the well-being of residents in various medical specializations [19-22], there has been little exploration of experiences of stress in residents working in an ICU. A single study focused on the effects of sleep deprivation in ICU rotation residents [23]. Evidence shows that individuals experiencing burnout have elevated resting HRs compared to healthy controls [24]. Another study found that first-year medical students have higher hemodynamic parameters (HR and blood pressure) than college undergraduates [25]. Despite the nearly universal inclusion of HR as a variable in biobehavioral studies, there has been little agreement regarding its effectiveness as a predictor of stress in the general population [26] and in residents [27].

The TILES (Tracking Individual Performance With Sensors)-2019 is a multimodal dataset collected from residents in the ICU via a multitude of instruments such as wearables, proximity sensors, and EMAs [28]. The authors of the dataset found patterns linking daily self-reported stress ratings to computer use habits within the ICU and resident job satisfaction to the frequency of interactions with attending physicians [29]. A study on a similar dataset that collected atypical event data from workers in the health care and aerospace industries reported that even minor negative events can significantly increase negative affect, anxiety, and stress [18]. The TILES-2019 dataset contains daily HR measurements, perceived stress reports, and the presence or absence of atypical events, making it an ideal source to study their effect on resident well-being.

### Objectives

In this study, our primary objective was to assess the use of average HR (AHR) as a physiological marker of psychological stress. In addition, we aimed to investigate the influence of atypical events on the relationship between AHR and perceived stress in ICU residents. Previous studies have shown mixed associations between HR indexes and stress [26,27], and at least one study has found that negative atypical events are strongly associated with higher self-reported stress [18]. However, no study has tested whether those events also change the concurrent physiological correlate. We used an exploratory approach and hypothesized that negative atypical events would significantly moderate the association between daily AHR and perceived stress. Specifically, we expected that (1) the strength of the relationship between AHR and stress would differ between standard days (no atypical events) and atypical event days and (2) negative atypical events would elevate perceived stress on average, with major work or life events producing larger increases than minor daily hassles.

Therefore, we used the EMA data provided by the TILES-2019 dataset to investigate differences in residents’ self-reported stress between standard and atypical event days to capture dynamic, day-to-day stressors. A linear mixed model (LMM) was used to analyze day-to-day variations in residents’ AHR to explain changes in stress response over the data collection period. The findings provide additional nuanced insights that explain the disparity in the literature regarding the predictive power of HR on perceived stress in resident physicians. Our results suggest that future biobehavioral studies should consider negative atypical events as significant data points.

## Methods

### Dataset

The TILES-2019 dataset consists of physiological and behavioral data collected from a cohort of 57 medical residents working in an ICU at the Los Angeles General Medical Center (formerly the Los Angeles County and University of Southern California Medical Center) [28]. The data collection efforts for the TILES-2019 study began in November 2019 and concluded in April 2020. Residents were invited to participate if they rotated through the ICU during the data collection period. In addition, they needed to be proficient in speaking and reading English and have access to an internet- and Bluetooth-enabled personal smartphone, a personal email account, and Wi-Fi at home. Briefly, over a 3-week rotation, including both workdays and nonworkdays, residents wore a Fitbit Charge 3 smartwatch that captured continuous HR data. Residents also used a custom smartphone app (the TILES study app) on their personal device to complete daily EMAs that assessed various features related to well-being, such as end-of-day stress, sleep, job performance, and job satisfaction. They underwent a 2-hour onboarding session where, among other topics, they were instructed in the use of the smartphone app and answering EMAs through their personal smartphone. The afternoon‑only prompt may be better described as a daily diary assessment rather than an EMA. To mark this distinction, we will henceforth refer to the daily stress item as a daily diary measure rather than an EMA prompt, although methodological sources often consider daily diary protocols to be a special variant within the broader EMA family [29-31].

The study assessed stress using a single item for which residents were prompted once a day on a fixed-interval design in the afternoon. Specifically, they were asked the following—“How would you rate your level of stress this afternoon?”—on a Likert scale from 1 to 7 [32]. Although this item is unvalidated, single-item Likert scale questions are frequently used in EMA research to keep participant burden low. A similar 7-item Likert scale was used by Janicki et al [33] in their study on acute stress in emergency medicine residents, and several recent EMA studies have similarly used a single, unvalidated item to capture stress or a closely related affective construct [34-37]. In addition, residents could specify whether an atypical event took place either during or outside their work shift. The overall compliance for Fitbit use and daily diary completion was 71% and 69.7%, respectively. In the study presented in this paper, a subset of items from the daily diary responses of residents was evaluated.

### Preprocessing

To facilitate the analysis of longitudinal trends in stress, the following preprocessing steps were implemented in pandas [38] to combine streaming HR readings from the Fitbit smartwatch with the daily survey responses.

First, Fitbit sampled HR data continuously at 5- to 10-second intervals. For a particular resident, we calculated the AHR on a given date (from midnight to 11:59 PM Pacific Standard Time) by taking the average of the HR values. At times, there were gaps in the Fitbit data, which we addressed using a quality control procedure.

Second, among health care professionals, stress can be attributed to incidents that occur both within and outside the hospital setting [39]. On a given day, residents could indicate whether they experienced an atypical event. If present, they could mark the atypical event as *positive* (eg, celebrating a birthday) or *negative* (eg, unexpected stressors at work). Negative atypical events could be further differentiated as days with either *daily hassles* or *negative work/life events*. We omitted days in which positive atypical events were reported as the frequency (4/136, 2.9% atypical event days) of positive events was too low to analyze separately.

Third, extreme working hours have been implicated as a pertinent precipitant of burnout and depressive symptoms in various health care and non–health care environments [40-42]. The effect of the number of hours worked on resident burnout has been explored and found to be significant [43]. We controlled for the number of hours worked in a day by calculating the difference between the start and end times of the work shift and including it as a covariate in the LMM. Some entries had missing start or end times for work shifts (not both); this was resolved by assuming the default 12-hour work shift as defined in the original TILES-2019 study [31].

Fourth, each set of daily diary responses corresponding to a resident was chronologically sorted. The day numbers corresponding to each resident’s service period in the ICU were included as repeated-measure variables in our LMMs.

Fifth, previous studies have determined that resident sex [38,44,45] and postgraduate year (PGY) [46] are significant factors that influence perceived stress. We used sex and PGY from baseline surveys as covariates in the LMM. Age was not considered as a covariate due to the limited range of age values in the study population.

### Quality Control

In biobehavioral studies using wearables, despite participants’ sincere attempts to adhere to protocols, gaps in recorded data are common due to multiple factors (eg, showering, charging, or forgetfulness) [47]. Incomplete physiological data generally result in inconclusive findings that cast doubt on the viability of wearables as effective ambulatory monitoring devices. To ensure accurate AHR calculations, the following quality control procedure was applied to each resident’s Fitbit data file:

1. For a given date, find the rows of data with a matching date time stamp.
2. Store the number of rows (N). As Fitbit samples data every 5 to 10 seconds, a resident can have an N value in the range of 8640 to 17,280.
3. To account for device inaccuracies, assume that an ideal resident must have 10,800 rows of data. This is a fair assumption as it is equivalent to a sampling rate of 1 reading every 8 seconds.
4. Divide N by 10,800 and multiply the result by 24 to derive the total number of hours for which the Fitbit was worn (H) on the given date.
5. For the given date, include the calculated AHR in the final dataset only if the value of H is above a threshold number of hours (Q). Return a NULL value otherwise.

On a given date, the total number of recorded HR values for a resident was divided by 10,800. This provided an estimate for the number of hours for which the Fitbit was genuinely worn assuming a sampling rate of 1 reading every 8 seconds. The computed AHR value for that day was considered valid only if the resident wore the Fitbit for at least a specified threshold amount of hours. Residents were filtered by empirically determining an ideal cutoff point for the number of *hours worn*.

As LMMs are designed to handle missing data, rows without self-reported stress (due to an incomplete EMA), AHR (due to noncompliance), or hours worked were retained. We did not observe any clear pattern for nonresponse to the daily survey, although no analysis was conducted to link missing data with self-reported stress.

### Data Analysis

A multiple-comparison analysis was conducted to compare the mean self-reported stress on standard days (when no atypical event was reported) and days with the aforementioned atypical event types.

We analyzed daily diary data using LMMs to account for both within-subject variance and between-subject variance. The LMM is appropriate for repeated observations with an unequal number of observations per participant where the outcome variable approximates a normal distribution [48,49]. Level 1 variables (within-subject variance) included daily atypical event, daily hours worked, AHR, and the interaction term between daily atypical event and AHR. Level 2 variables (between-subject variance) included sex and PGY. All continuous predictors (AHR and hours worked) were person mean centered before analysis. The number of calendar days elapsed since each resident’s study day was treated as a repeated measure nested within persons. We used an autoregressive covariance structure to account for serial correlation across consecutive days within individuals. A random intercept for residents (covariance type=variance components) allowed baseline stress levels to vary across individuals. Fixed effects were estimated via maximum likelihood with Satterthwaite df. Statistical significance was defined as *P*<.05. To assess whether the AHR-stress association was moderated by the presence of atypical events, we fit 2 hierarchical mixed models. Model 1 contained only main effects (AHR, hours worked, atypical event, and covariates). Model 2 added the atypical event × AHR interaction term. For our secondary analysis of event severity (model 3), we replaced the binary atypical event variable with a categorical variable with 3 levels (standard day, daily hassle, and negative work or life event), where standard day was used as the reference category. All statistical analyses were conducted using SPSS Statistics for Windows (version 29.0.0.0; IBM Corp).

### Ethical Considerations

The original TILES-2019 study was conducted in accordance with the University of Southern California Institutional Review Board approval (study ID HS-19-00606) [31]. This study was conducted using the publicly available and deidentified TILES-2019 dataset and was exempt from institutional review board review. Participants in the original TILES study provided consent for the collection and use of their data for secondary analysis as documented by the original study authors as part of their data use agreement. For their participation, residents received weekly gift cards and were allowed to keep the Fitbit device.

## Results

### Quality-Controlled Dataset

**Figure 1:**
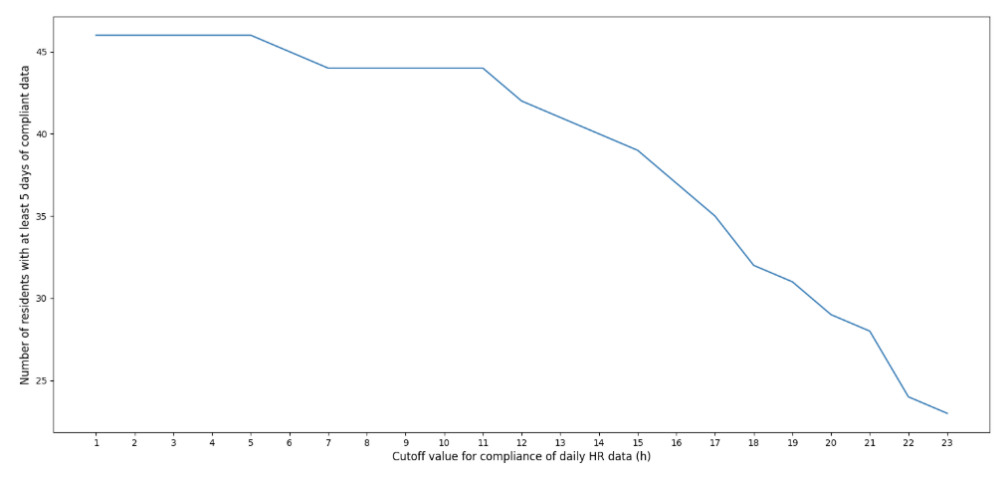
Empirical determination of the quality control threshold for Fitbit heart rate (HR) data. A resident’s HR data for a day were considered compliant only if they wore the Fitbit for at least 11 hours. Increasing the strictness of the hours worn threshold (x-axis) decreased the number of compliant residents (y-axis).

On the basis of the defined quality control procedure, Figure 1 shows the number of residents with at least 5 days (approximately 25% of the study duration) of compliant HR data while varying the threshold number of total available hours of HR data. It was empirically determined that 11 hours of available HR data was a cutoff that balanced data quality with exclusion of residents for noncompliance. The final dataset comprised 44 residents.

### Demographics

Table 1 shows demographic information, with most residents identifying as Asian individuals (26/44, 59%), followed by White (11/44, 25%) and Hispanic or Latino individuals (3/44, 7%). A smaller proportion identified as Black individuals, multiethnic, or other or opted not to disclose their race or ethnicity (1/44, 2% each). The vast majority of residents (35/44, 80%) were from the internal medicine program, whereas 16% (7/44) were from emergency medicine, and 5% (2/44) were from the internal medicine or pediatrics program. The distribution across PGYs was relatively balanced, with 36% (16/44) in their first year, 34% (15/44) in their second year, and 30% (13/44) in their third year of training. Male individuals constituted most of the sample at 64% (28/44).

**Table 1:** Demographic information of residents in the quality-controlled dataset (N=44)^a^.

Characteristic			Values
Age (y), mean (SD)			29.13 (2.03)
**Race or ethnicity, n (%)**			
	Asian	26 (59)	
	Black	1 (2)	
	Hispanic or Latino	3 (7)	
	Multiethnic	1 (2)	
	White	11 (25)	
	Prefer not to say	1 (2)	
	Other	1 (2)	
**Specialty, n (%)**			
	Internal medicine	35 (80)	
	Emergency medicine	7 (16)	
	Meds or pediatrics	2 (5)	
**Postgraduate year, n (%)**			
	1 (intern)	16 (36)	
	2 (junior)	15 (34)	
	3 (senior)	13 (30)	
**Sex, n (%)**			
	Male	28 (64)	
	Female	16 (36)	

^a^The final sample size constitutes participants with at least 5 days of heart rate data.

### Descriptive Statistics of Quality-Controlled AHR

After applying the ≥11-hour wear time threshold, 710 days of HR data were retained for analysis, corresponding to 77% (44/57) of the enrolled residents. The daily AHR across these observations was 76.8 (SD 17.0) beats per minute (bpm). The distribution was approximately symmetrical (median 76, IQR 64-87 bpm; Table 2). Outlier values were rare, with the lowest daily mean recorded at 34 bpm and the highest at 202 bpm. These descriptive statistics fall within expected physiological ranges for adults.

**Table 2:** Descriptive statistics of quality-controlled average heart rate (AHR)^a^.

Statistic	Daily AHR (bpm^b^)
Values, mean (SD)	76.8 (17.0)
Values, median (IQR; range)	76 (64-87; 34-202)

^a^AHR was computed as the mean of continuous 5- to 10-second Fitbit heart rate data on each day with the ≥11-hour wear time.

^b^bpm: beats per minute.

### Types of Negative Atypical Events

Residents experienced mostly standard days (n=588), with fewer occurrences of negative work or life events (n=77) and daily hassles (n=55). Multiple-comparison analysis using Bonferroni corrections showed significant differences in perceived stress across atypical event types. Specifically, the mean difference in perceived stress between work or life event days and standard days was 1.99 (*P*<.001), whereas the mean difference between daily hassle days and standard days was 0.941 (*P*<.001). Furthermore, perceived stress on work or life event days was significantly higher than perceived stress on daily hassle days, with a mean difference of 1.05 (*P*<.001).

### LMM Results

**Figure 2:**
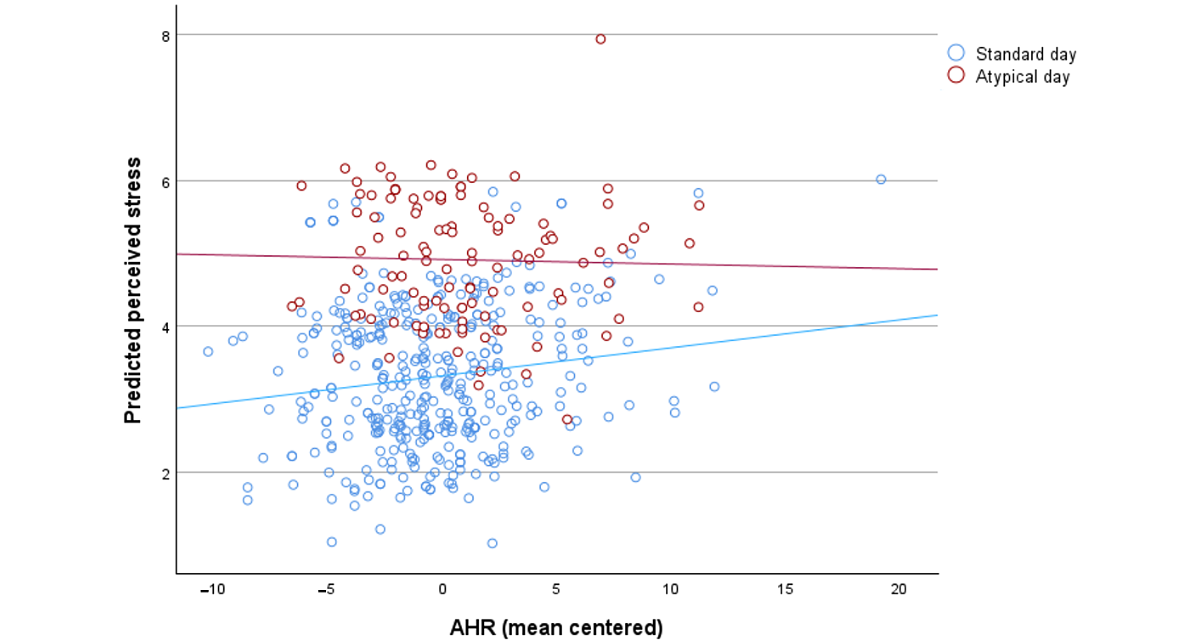
Interaction effect between presence or absence of a negative atypical event day and mean-centered average heart rate (AHR) on perceived stress.

Model 1 showed no significant association between AHR and perceived stress (β=0.016, SE 0.014; 2-tailed t_424_=1.15; *P*=.25), whereas the number of hours worked (β=0.415, SE 0.087; t_17.4_=4.79; *P*<.001) and the presence of a negative atypical event (β=1.36, SE 0.22; t_30.3_=6.27; *P*<.001) were significantly positively associated with perceived stress (Table 3). Model 2 included the atypical event × AHR interaction, which was significant (β=−0.076, SE 0.032; t_435_=−2.35; *P*=.02), indicating that AHR was associated with higher stress on standard days (β=0.032, SE 0.016; t_415_=2.09; *P*=.04) but not on negative atypical event days (Table 4 and Figure 2).

**Table 3:** Model 1, a linear mixed model assessing the relationship between mean-corrected average heart rate (AHR) and perceived stress as measured using a daily diary^a^.

Parameter	Perceived stress estimate (β; 95% CI)	*t* test (*df*)^b^	*P* value^c^
Intercept	3.21 (2.60 to 3.81)	10.7 (45.6)	*<.001* ^c^
Female sex (reference=male)	0.129 (−0.482 to 0.740)	0.427 (40.0)	.67
PGY^d^ 1 (reference=PGY 3)	0.390 (−0.340 to 1.12)	1.08 (40.7)	.29
PGY 2 (reference=PGY 3)	−0.279 (−1.01 to 0.454)	−0.770 (39.1)	.45
Hours worked^e^	0.415 (0.233 to 0.598)	4.79 (17.4)	*<.001*
AHR^e^	0.016 (−0.011 to 0.043)	1.15 (424)	.25
Negative atypical event (reference=standard day)	1.36 (0.918 to 1.80)	6.27 (30.3)	*<.001*

^a^Variables assessed include person mean–centered AHR, presence of atypical events, daily hours worked, resident sex, and postgraduate year. No significant association was found between AHR and daily perceived stress. Longer work hours and presence of a negative atypical event were associated with higher perceived stress, whereas sex and postgraduate year were not.

^b^Wald *t* statistic from SPSS MIXED procedure (Satterthwaite df).

^c^Italicized values indicate statistical significance (*P*<.05, 2-tailed).

^d^PGY: postgraduate year.

^e^Values are mean centered.

**Table 4:** Model 2, a linear mixed model assessing the interaction effect between atypical event and mean-centered average heart rate (AHR) on perceived stress^a^.

Parameter	Perceived stress estimate (β; 95% CI)	*t* test (*df*)^b^	*P* value^c^
Intercept	3.22 (2.62 to 3.83)	10.7 (45.7)	*<.001* ^c^
Female sex (reference=male)	0.117 (−0.498 to 0.731)	0.383 (40.3)	.70
PGY^d^ 1 (reference=PGY 3)	0.403 (−0.330 to 1.14)	1.11 (40.9)	.27
PGY 2 (reference=PGY 3)	−0.289 (−1.03 to 0.448)	−0.792 (39.5)	.43
Hours worked^e^	0.418 (0.237 to 0.599)	4.87 (16.9)	*<.001*
AHR^e^	0.032 (0.002 to 0.063)	2.09 (415)	*.04*
Negative atypical event (reference=standard day)	1.40 (0.959 to 1.84)	6.49 (30.7)	*<.001*
Atypical event × AHR^e^	−0.076 (−0.140 to −0.013)	−2.35 (435)	*.02*

^a^Same as model 1 plus the interaction term. AHR was significantly associated with stress on standard days. This association was attenuated on days with a negative atypical event, as indicated by the significant negative interaction term.

^b^Wald *t* statistic from SPSS MIXED procedure (Satterthwaite df).

^c^Italicized values indicate statistical significance (*P*<.05, 2-tailed).

^d^PGY: postgraduate year.

^e^Values are mean centered.

This moderating effect of negative atypical events persisted in our secondary analysis (model 3) on both daily hassle and negative work or life event days (β=−1.02 and −0.63, respectively), but the interaction term between these and AHR did not reach statistical significance (*P*=.05 and .11, respectively; Table 5). Both daily hassle (β=0.689, SE 0.268; t_52.2_=2.57; *P*=.01) and negative work or life events (β=1.71, SE 0.219; *t_39.0_*=7.84; *P*<.001) were significantly associated with increased stress compared to standard days, with negative work or life events exerting a more pronounced effect (Table 5).

**Table 5:** Model 3, a linear mixed model assessing the interaction effect between severity of negative atypical event and mean-centered average heart rate (AHR) on perceived stress^a^.

Parameter	Perceived stress estimate (β; 95% CI)	*t* test (*df*)^b^	*P* value^c^
Intercept	3.20 (2.61 to 3.79)	10.9 (43.8)	*<.001* ^c^
Female sex (reference=male)	0.146 (−0.456 to 0.748)	0.490 (38.8)	.63
PGY^d^ 1 (reference=PGY 3)	0.473 (−0.245 to 1.19)	1.33 (39.4)	.19
PGY 2 (reference=PGY 3)	−0.283 (−1.00 to 0.438)	−0.794 (37.9)	.43
Hours worked^e^	0.369 (0.199 to 0.539)	4.66 (13.5)	*<.001*
AHR^e^	0.036 (0.006 to 0.066)	2.33 (407)	*.02*
Daily hassle (reference=standard day)	0.689 (0.151 to 1.23)	2.57 (52.2)	*.01*
Negative work or life event (reference=standard day)	1.71 (1.27 to 2.16)	7.84 (39.0)	*<.001*
Daily hassle × AHR^e^ (reference=standard day)	−0.102 (−0.206 to 0.002)	−1.93 (448)	.05
Negative work or life event × AHR^e^ (reference=standard day)	−0.063 (−0.140 to 0.014)	−1.61 (439)	.11

^a^Negative atypical events were further classified into a 3-level categorical factor (standard day [reference], daily hassle, and major work or life event) with corresponding AHR interaction terms. Both minor and major negative events increased perceived stress and attenuated the positive association between AHR and perceived stress, although interaction terms did not reach statistical significance.

^b^Wald *t* statistic from SPSS MIXED procedure (Satterthwaite df).

^c^Italicized values indicate statistical significance (*P*<.05, 2-tailed).

^d^PGY: postgraduate year.

^e^Values are mean centered.

## Discussion

### AHR-Stress Association

The significant association between higher daily AHR and increased levels of daily stress among ICU residents on standard days demonstrates a connection between physiological parameters and psychological well-being in a subpopulation exposed to numerous acute and chronic stressors. Our finding underscores the value of leveraging AHR as an objective, noninvasive, and scalable measure to understand and monitor stress levels in medical residents. This is of particular importance given that physician stress is reported to not only negatively impact their health but also diminish patient-perceived quality of care and increase the risk of adverse medical events [50,51].

### Relation to Previous Literature

Previous studies have examined HR response to stressful stimuli in medical residents. Tendulkar et al [52] reported a significant increase in AHR in a group of surgical residents on a 24-hour call shift regardless of year of training compared to their AHR when off clinical responsibilities. Similarly, in a 2021 prospective observational study involving a cohort of emergency medicine residents, Janicki et al [33] found a significant increase in AHR while residents were clinically engaged compared to baseline HR measured during didactics. The study also reported a significant increase in subjectively recorded stress levels during the clinical period; however, it did not assess the association between mean HR and subjective stress directly [33].

Previous literature among the general population examining the relationship between HR and perceived stress as measured using EMA has been mixed, with some studies finding no association between HR and stress; some identifying positive associations; and others finding context-dependent positive associations, such as in the presence of chronic stress and posttraumatic stress disorder [26]. Overall, the current literature suggests that HR can be a physiological marker of stress in residents but its effectiveness and the nature of the association may depend on specific contexts and individual differences that moderate the relationship between HR and perceived stress.

### Atypical Events as Moderators of Stress and AHR

One such moderator may be the presence of atypical events. In line with a previous study on the effects of atypical events on perceived stress in a combined population of nurses and aviation workers, we found that the presence of negative atypical events regardless of severity led to a significant increase in perceived stress when compared to a standard day [18]. Our study additionally suggests that the positive association between AHR and stress may only occur on standard days. Including days with negative atypical events attenuates this relationship to the point of statistical nonsignificance. This effect may persist on both severe atypical event days, defined as work or life events, as well as minor atypical event days, defined as daily hassles. Specifically, our analysis did not find a differential moderating effect of atypical event severity on the relationship between AHR and perceived stress. Both minor and severe atypical event days appear to attenuate the positive relationship between AHR and stress observed on standard days, albeit these effects not being statistically significant, likely due to our limited sample size.

The physiological basis underlying the moderating effect of atypical events is unclear. One possible contributory factor may involve a biological habituation process in response to frequent exposure to stressors, resulting in a blunting in physiological response while psychological recall and rating of the stressors remain intact. Stress exposure engages multiple endogenous stress-reactive systems, including the sympatho-adrenomedullary (SAM) system [53-56]. Activation of the SAM system occurs first and initiates the release of catecholamines that increase HR, blood pressure, and glucose production to prepare the body for a *fight or flight* response [54]. While this acute stress response is adaptive in the short term, frequent activation of the SAM system may result in a blunted HR response without concomitant change in subjective stress ratings [57]. The implications of this blunted physiological response is highlighted by evidence from numerous prospective studies that have linked reduced HR reactivity to stress with adverse health outcomes, including heightened risks of obesity, depression, anxiety, and posttraumatic stress disorder symptoms; increased illness frequency; musculoskeletal pain; and declines in both cognitive and physical health [56,58,59].

Our study suggests that the psychological perception of stress may be masked if only AHR is considered, leading to a potential underestimation of stress levels in these critical periods. Incorporating negative atypical events as a moderating factor may result in more accurate and context-sensitive interpretations of AHR data. This is crucial for understanding how stress manifests in residents working in high-pressure environments such as ICUs, which may allow for the development of better targeted interventions and, ultimately, the improvement of well-being for residents and their patients.

### Limitations

This study has several limitations. The sample size was relatively small and drawn from a single institution, which limits statistical power and the strength of our conclusions. The TILES-2019 study did not collect data on potential confounding factors that could contribute to the hyper- or hypoactivation of stress response systems, such as smoking, caffeine intake, presence of medical conditions, or engagement in physical activity during and outside of clinical hours. In addition, the lack of diversity and skew toward male individuals in our demographic composition may limit the generalizability of our findings to underrepresented ethnic groups or female individuals, who may experience stress differently or have different physiological responses to stress. Importantly, our outcome variable was measured using a once-daily, unvalidated Likert-scale item. While this necessarily limits content validity and reliability estimation, it remains a pragmatic compromise for high-burden participants such as medical residents and is widely used in contemporary EMA research, having been adopted in multiple recent peer-reviewed studies [33-37]. Finally, we note that positive atypical events were excluded from our analyses due to their low number; however, positive events can also affect HR and stress in significant ways.

### Conclusions

We demonstrated the utility of AHR as a physiological marker for stress among ICU residents. The presence of atypical events attenuates this relationship, suggesting that AHR alone may not fully capture psychological stress during these critical periods and that incorporating atypical events as a moderating factor should be considered for more accurate stress assessment. The use of EMA was key to this as it allowed us to capture fluctuations in stress on a more granular level that would be missed through infrequent or more retrospective assessment. Further investigation should determine whether this moderating effect can be replicated in more diverse cohorts of residents, assess its generalizability to broader populations, and control for additional confounding variables that may influence these associations.

## Abbreviations

AHR: average heart rate
bpm: beats per minute
EMA: ecological momentary assessment
HR: heart rate
ICU: intensive care unit
LMM: linear mixed model
PGY: postgraduate year
SAM: sympatho-adrenomedullary
TILES: Tracking Individual Performance With Sensors

## Data Availability

The datasets generated or analyzed during this study are available in the TILES (Tracking Individual Performance With Sensors) repository [60].
